# Measure of Internalized Sexual Stigma for Lesbians and Gay Men (MISS-LG) in Taiwan: Psychometric Evidence from Rasch and Confirmatory Factor Analysis

**DOI:** 10.3390/ijerph182413352

**Published:** 2021-12-18

**Authors:** Cheng-Fang Yen, Yu-Te Huang, Marc N. Potenza, Tzu-Tung Tsai, Chung-Ying Lin, Hector W. H. Tsang

**Affiliations:** 1 Department of Psychiatry, School of Medicine, College of Medicine, Kaohsiung Medical University, Kaohsiung 802, Taiwan; chfaye@cc.kmu.edu.tw; 2 Department of Psychiatry, Kaohsiung Medical University Hospital, Kaohsiung 802, Taiwan; 3 College of Professional Studies, National Pingtung University of Science and Technology, Pingtung 91201, Taiwan; 4 Department of Social Work and Social Administration, The University of Hong Kong, Hong Kong RM543, China; Yuhuang@hku.hk; 5 Departments of Psychiatry and Neuroscience and the Child Study Center, School of Medicine, Yale University, New Haven, CT 06511, USA; marc.potenza@yale.edu; 6 Connecticut Council on Problem Gambling, Wethersfield, CT 06109, USA; 7 Connecticut Mental Health Center, New Haven, CT 06519, USA; 8 Wu Tsai Institute, Yale University, New Haven, CT 06510, USA; 9 Department of Neurology, E-Da Hospital, I-Shou University, Kaohsiung City 82445, Taiwan; 10 Institute of Allied Health Sciences, College of Medicine, National Cheng Kung University, Tainan 701, Taiwan; 11 Department of Occupational Therapy, College of Medicine, National Cheng Kung University, Tainan 701, Taiwan; 12 Department of Public Health, National Cheng Kung University Hospital, College of Medicine, National Cheng Kung University, Tainan 701, Taiwan; 13 Biostatistics Consulting Center, National Cheng Kung University Hospital, College of Medicine, National Cheng Kung University, Tainan 701, Taiwan; 14 Department of Rehabilitation Sciences, Faculty of Health and Social Sciences, The Hong Kong Polytechnic University, Hung Hom, Kowloon, Hong Kong 999077, China; Hector.Tsang@polyu.edu.hk

**Keywords:** factor analysis, psychometrics, psychological well-being, Rasch, sexual minority, stigma

## Abstract

Internalized sexual stigma may hamper the development of self-identity, social interaction, and intimate relationship in lesbian, gay, and bisexual (LGB) individuals. The Measure of Internalized Sexual Stigma for Lesbians and Gay Men (MISS-LG) is an important instrument assessing internalized sexual stigma for LGB individuals. However, its psychometric properties have not been examined in LGB populations outside the Italian community. The present study used advanced psychometric testing to evaluate the traditional Chinese version (the MISS-LG) among LGB individuals in Taiwan. LGB individuals (500 male, 500 female) participated in this study and completed the MISS-LG, HIV and Homosexuality Related Stigma (HHRS), and Acceptance and Action Questionnaire-II (AAQ). Confirmatory factor analysis results confirmed the three-factor structure of the MISS-LG; however, two items for males and one item for females had somewhat low factor loadings on the sexuality factor. Each MISS-LG factor was found to be unidimensional in Rasch results, except for the slight misfit in Item 3 and concurrent validity of the MISS-LG was supported by the positive correlations with HHRS and the AAQ. The Chinese MISS-LG has relatively satisfactory psychometric properties. However, further research is needed to investigate the reasons for problematic fitting of several items.

## 1. Introduction

A wide range of social stigma derived from heterosexism toward lesbian, gay, and bisexual (LGB) individuals prevails worldwide [1]. Structural stigma, including bans/restrictions on same-sex relationships and health care disparities among LGB individuals [2,3,4,5,6], may promote other stigma such as bullying and hate crimes against LGB individuals [7], and disapprobation of LGB cultures [8] may impact the identities, behaviors, health, and relationships of LGB individuals profoundly. Internalized sex stigma, or internalized homonegativity, refers to the process whereby LGB individuals perceive and internalize societal messages toward gender and sex—often unconsciously, as part of their self-image [9]. Negative feelings toward themselves may threaten their psychological well-being [10] and increase risks of mental health problems [11,12] among LGB individuals. LGB individuals with high internalized sexual stigma may avoid disclosing their sexual orientation to others [12] to protect themselves from harm [13]; however, it may damage LGB individuals’ relationship well-being [14] and reduce social support. LGB individuals may use alcohol and addictive substance use to cope with the stress-related to internalized sexual stigma [15,16]. Research has found that internalized sexual stigma was associated with practicing HIV at-risk sexual behaviors, such as condomless sex, multiple sex partners, and unprotected anal sex among gay men [12,15,17]. Internalized sexual stigma may also deter the LGB individuals from seeking medical services. For example, a previous study on Chinese men who have sex with men indicated that internalized homophobia was a major barrier to accessing human immunodeficiency virus (HIV) prevention and care services [18].

Young adulthood is a phase of the life span from adolescence to full-fledged adulthood where the individuals become more independent and explore various life possibilities [19]. LGB individuals may experience multiple dimensions of social stigma during their adolescence and internalize sexual stigma; internalized sexual stigma may be intensified in early adulthood when they become more independent and expand aspects of life. Internalized sexual stigma may hamper the development of self-identity, social interaction, and intimate relationships in LGB individuals during early adulthood. There is an urgent need to assess the internalized sexual stigma for LGB individuals during early adulthood. Therefore, a standardized instrument assessing internalized sexual stigma with good psychometric properties should be developed.

A review study identified six scales that were developed for measuring internalized sexual stigma and had the reports of psychometric properties [20]. Among them, the Reactions to Homosexuality Scale [21], Internalized Homonegativity Inventory [22], and Internalized Homophobia Scale [23] measured internalized sexual stigma in gay men; the Lesbian Internalized Homophobia Scale [24] measured internalized sexual stigma in lesbians; and the subscale of the Lesbian, Gay, and Bisexual Identity Scale [25] and Measure of Internalized Sexual Stigma for Lesbians and Gay men (MISS-LG) [26] measured internalized sexual stigma in lesbians and gay men.

The MISS-LG was developed using robust procedures, including (i) construct (i.e., internalized sexual stigma) definition; (ii) scale design; (iii) pilot testing; (iv) measure purification and scale administration; and (v) construct validation verification. Several rounds of psychometric testing with different methods (including content validity checking, cognitive interviewing, exploratory factor analysis, and confirmatory factor analysis) were applied to the MISS-LG; finally, the MISS-LG was verified to be a three-factor structure (i.e., sexuality, identity, and social discomfort) with 17 items [26]. The feature of the MISS-LG is that this scale has two versions, one for males and another for females with the same three first-order factors, the same number of items, and Likert-type scale with some different item contents [26]. For example, the sexuality factor of the MISS-LG contains the item “If you are gay, it is better to have an active sexual role” in the version for gay and bisexual men only and the item “All lesbian women end up isolated and alone” in the version for lesbian and bisexual women only. As Szymanski et al. [27] noted, there are significant differences in the ways that internalized sexual stigma is experienced for sexual minority men and women, and it is important to identify manifestations that have some common features and specificities for lesbian women and gay men. Therefore, the MISS-LG will help capture gender-specific characteristics of internalized sex stigma.

Although the MISS-LG was developed using a robust procedure and has been cited more than 120 times since its release, the current literature provides little information on its psychometric properties. To the best of our knowledge, the psychometric properties of the MISS-LG have only been reported in one study [26], in the manuscript reporting the development of the MISS-LG. In the manuscript [26], study participants were Westerns, and therefore, psychometric properties of the MISS-LG should be evaluated in other populations (e.g., Eastern ones like in Taiwan) to evaluate its generalizability beyond WEIRD (White, educated, industrialized, rich, and democratic) groups [28]. Indeed, prior research has suggested the psychometric properties of an instrument should be accumulated for scientific reasons [29].

Tolerance to sexual minorities in Taiwan has outpaced that in China, Japan, and South Korea over the past two decades [30]. In May 2017, Taiwan’s Council of Grand Justices announced that the current Civil Code that barred same-sex relationships was a violation of human rights to equality and was unconstitutional. This announcement encouraged many LGB individuals in Taiwan. However, the results of the vote for the Same-Sex Marriage Referendums in Taiwan released on November 24, 2018, indicated that over 70% of voters opposed same-sex marriage defined in the Civil Code [31]. Although the Taiwanese government legalized same-sex relationships outside the Civil Code in May 2019 [32], unfavorable attitudes and unfriendly behaviors appear to remain rooted in many Taiwanese people’s mindset and difficult to change, especially for those with strong traditional Chinese ethics [33] or Christian faith [30]. Many LGB individuals are likely to experience and be impacted by these unfavorable attitudes and unfriendly behaviors and gradually develop internalized sexual stigma.

In order to supplement the psychometric evidence of the MISS-LG in non-Western societies, the present study aimed to use two types of psychometric testing (i.e., classical test theory and item response theory) [34,35] to evaluate the MISS-LG among LGB individuals in Taiwan. Specifically, the classical test theory in the present study adopts the confirmatory factor analysis (CFA) to verify whether the three-factor structure of the MISS-LG can be replicated among LGB individuals in Taiwan. Classical test theory was also used to examine the concurrent validity of the MISS-LG after its three-factor structure was confirmed in the present sample. For item response theory, Rasch modeling [36,37] was applied to examine whether the each of three factors had a unidimensional feature.

We also examined the concurrent validity of the MISS-LG by testing the correlations between internalized sex stigma and perceived social stigma attitudes toward homosexuality and psychological flexibility. We hypothesized that the MISS-LG factors would have significant correlations with perceived social stigma attitudes toward homosexuality and psychological flexibility because of the following reasons: (1) as internalized sex stigma is the result whereby LGB individuals perceive and internalize social stigma toward sexual minority, internalized sex stigma and perceived social stigma attitudes toward homosexuality should be positively associated; (2) individuals with a higher level of psychological flexibility may cope better with unfriendly environments that may prevent generation of internalized stigma [38].

## 2. Materials and Methods

### 2.1. Participants and Procedure

We recruited participants by posting online advertisements on social media including Facebook, Twitter, and LINE messaging app (Line Corporation, Tokyo, Japan), the Bulletin Board System, and the home pages of three health promotion and counseling centers for LGB individuals from August 2018 to July 2019. The recruitment criteria were individuals who identified their sexual orientation as homosexual or bisexual, aged between 20 and 30 years, and lived in Taiwan. Anyone interested in participating in this study was to phone the research assistants. Research assistants assessed the eligibility of potential participants based on study criteria, explained the study aims and procedures to them, and scheduled times for completing the study questionnaires individually in the study room. The research assistants evaluated participants face-to-face in the study room to determine whether they had impaired intellect or evidence of alcohol and substance use that might interfere with understanding the study’s purpose or method of completing the questionnaire; if they had, they were excluded from the study. In total, 1000 participants (500 males and 500 females) participated. No participants were excluded. Informed consent was obtained from all participants. This study was approved by the Institutional Review Board of Kaohsiung Medical University Hospital—KMUHIRB-F(II)-20180018.

### 2.2. Measures

#### 2.2.1. The MISS-LG

The MISS-LG contains 17 items regarding internalized sexual stigma for lesbian women and gay men [26]. The MISS-LG has two versions, one for lesbian women and another for gay men, with the same factor structure (i.e., three factors of sexuality, identity, and social discomfort). The identity factor (5 items) indicates an enduring propensity to have a negative self-attitude as homosexual and to consider sexual stigma as a part of self-identity (e.g., “If it were possible, I would do anything to change my sexual orientation”). The social discomfort factor (7 items) reflects fear of public identification as a lesbian woman or gay man in the social context and disclosure in private and professional life (e.g., “When I feel attracted to another lesbian/gay man, I hope no one realizes it”). The sexuality factor (5 items) indicates the pessimistic attitudes toward the quality and duration of intimate relationships in lesbians and gay men (e.g., “Lesbians/gay men can only have flings/one-night stands”) and the negative conception of gay or lesbian sexual behaviors (e.g., “When I have sex with a woman/man, I feel awkward”). All MISS-LG items are rated on a five-point Likert type scale with the descriptors of strongly disagree (score 1), disagree (score 2), neither disagree nor agree (score 3), agree (score 4), and strongly agree (score 5); therefore, a higher MISS-LG score indicates higher levels of internalized sexual stigma. The MISS-LG has demonstrated satisfactory psychometric properties [26]. The internal consistency of MISS-LG was acceptable to excellent in the present sample (McDonald’s omega = 0.67 for sexuality in male participants; = 0.87 for identity in male participants; = 0.90 for social discomfort in male participants; = 0.64 for sexuality in female participants; = 0.87 for identity in female participants; and = 0.91 for social discomfort in female participants).

The MISS-LG has been translated into a Chinese version for Taiwanese LGB individuals using standard forward-, backward-, and pretest-step methods [39]. First, the original version was translated into a traditional Chinese version by one bilingual translator. Next, the traditional Chinese version was back-translated into English by another bilingual translator. Finally, the original version was compared with the back-translation. If discrepancies arose in the back-translation, translators worked cooperatively to make corrections in the final traditional Chinese version. We further invited three experts in the field of sexuality study to examine the adequacy of the questionnaire.

#### 2.2.2. Measures Used for Concurrent Validity of the MISS-LG

We used two measures for examining the concurrent validity of the MISS-LG. The first measure was the HIV and Homosexuality Related Stigma (HHRS) [40]. We adopted the 12 items on the HHRS-Homosexuality subscale measuring the stigma attitudes toward homosexuality that LGB individuals perceive from their families. The items are rated on a four-point Likert type scale with the descriptors of strongly disagree (score 1), disagree (score 2), agree (score 3), and strongly agree (score 4); therefore, a higher HHRS-Homosexuality score indicates a higher level of perceived stigma related to homosexuality from families. The HHRS-Homosexuality had satisfactory psychometric properties in prior research [39] and in the present sample (e.g., McDonald’s omega = 0.95 in male participants; and = 0.94 in female participants).

The second measure is the Acceptance and Action Questionnaire-II (AAQ) [41]. The AAQ contains 7 items asking an individual’s psychological flexibility. All the AAQ items are rated on a seven-point Likert type scale with the descriptors of completely disagree (score 1), almost always disagree (score 2), rarely agree (score 3), sometimes agree (score 4), usually agree (score 5), almost always agree (score 6), and completely agree (score 7); therefore, a higher AAQ score indicates a lower level of psychological flexibility. The AAQ had satisfactory psychometric properties in prior research [41,42] and in the present sample (e.g., McDonald’s omega = 0.94 in male participants; =0.95 in female participants).

### 2.3. Sociodemographic and Sexual Orientation Factors

Data were collected regarding the participants’ gender (male vs. female), age, education level (high school or below vs. college or above), sexual orientation (“Do you identify yourself as a gay/lesbian or bisexual?”), paternal and maternal education, and sexual orientation known by family, friends, and online friends (no or few vs. many or a great quantity).

### 2.4. Data Analysis

Participants’ characteristics and MISS-LG item scores were first analyzed using descriptive statistics. Then, the distributions of the MISS-LG items were checked (where absolute skewness < 3 and absolute kurtosis < 10 indicate normal distribution [43]) before psychometric analyses were conducted. The psychometric testing of the MISS-LG involved three parts. First, CFA with a diagonally weighted least-squares estimator was used to examine the three-factor structure of the MISS-LG. Accordingly, factor loadings derived from the CFA were calculated with a value >0.3 indicating acceptability [44]. In addition, the CFA was examined using several fit indices to determine whether the three-factor structure was supported. The fit indices were the comparative fit index (CFI) > 0.95, Tucker–Lewis index (TLI) > 0.95, root mean square error of approximation (RMSEA) < 0.06, and standardized root mean square residual (SRMR) < 0.08 [45]. Second, Rasch analysis with a partial credit model was used to examine whether the items embedded in the MISS-LGB factors belonged to their constructs. Specifically, infit mean square (MnSq) and outfit MnSq were used to decide whether an item fit with its construct. When both infit and outfit MnSq of an item was between 0.5 and 1.5, this item was considered to be in its embedded construct [46]. Item difficulty was also calculated from the Rasch analysis. Third, concurrent validity of the traditional Chinese version of the MISS-LG was examined using the correlations between MISS-LG factors and other measures (i.e., HHRS-Homosexuality and AAQ).

All psychometric testing for the MISS-LG was conducted separately for male and female participants because of the different gender versions. All statistical analyses were conducted using R software (Boston, MA, USA) (the CFA was done with the use of lavaan package version 0.6–9; https://lavaan.ugent.be/index.html, accessed on 12 December 2021), except for the Rasch analyses. The Rasch analyses were done using the WINSTEPS (WINSTEPS, Chicago, IL, USA) (https://www.winsteps.com, accessed on 12 December 2021).

## 3. Results

The participants were 500 males (mean age = 24.80 years; SD age = 2.91; age range = 20–30) and 500 females (mean age = 24.45 years; SD age = 3.06; age range = 20–30). Most participants had obtained a college diploma or college degree (87.2% in male participants and 91.0% in female participants). Nearly three-fourths of male participants (73.0%) and slightly less than half of the female participants (41.0%) were homosexual. Other characteristics, including parental educational levels and whether participants’ sexual orientation was known by others, are presented in Table 1.

Table 2 reports the item properties and CFA results for the MISS-LG. All items were normally distributed or nearly normally distributed (skewness = −02 to 1.97, kurtosis = −1.25 to 3.87 for males; skewness = −0.11 to 3.13, kurtosis = −0.96 to 9.77 for females). Most items had an acceptable factor loading (i.e., >0.3) with the exceptions of items 8 (“I do not believe in love between homosexual individuals”; factor loading = 0.270 for males) and 11 (“If you are gay, it is better to have an “active” sexual role”; factor loading = 0.167 for males and “Lesbians can only have flings/one-night stands”; factor loading = 0.299 for females). Regarding the entire factor structure, the MISS-LG demonstrated a good fit with the three-factor structure by the support of excellent fit indices. For males, the fit indices were chi-square (df) = 232.47 (116), *p*-value < 0.001, CFI = 0.985, TLI = 0.983, RMSEA (90% CI) = 0.045 (0.036, 0.053), and SRMR = 0.062 or females, the fit indices were chi-square (df) = 186.62 (116), *p*-value < 0.001, CFI = 0.987, TLI = 0.985, RMSEA (90% CI) = 0.035 (0.025, 0.044), and SRMR = 0.056.

Results from Rasch analyses (Table 3) indicate that the difficulty range of the MISS-LG sexuality factor was between 0.97 and −1.03 for males and between 0.60 and −0.66 for females. The difficulty range of the MISS-LG identity factor was between 1.12 and −1.01 for males and between −1.12 and 0.85 for females. The difficulty range of the MISS-LG social discomfort was between −0.45 and 0.36 for males and between −1.29 and 0.93 for females. Moreover, almost all the items had good fit statistics in both infit and outfit MnSq with the exceptions of items 3 (“I’m worried to understand whether I like women”; outfit MnSq = 1.53) and 17 (“Effeminate gay men annoy me”; outfit MnSq = 1.51) for males.

Finally, the concurrent validity of the MISS-LG is shown in Table 4 with both HHRS-Homosexuality and AAQ measures. The correlations between the three factors of MISS-LG were in strong magnitude (*r* = 0.752 to 0.937 for males; = 0.640 to 0.784 for females; all *p*-values < 0.001). Moreover, the three MISS-LG factors were positively associated with HHRS-Homosexuality scores (*r* = 0.220 to 0.256 for males; = 0.190 to 0.384 for females; all *p*-values < 0.001) and AAQ scores (*r* = 0.323 to 0.427 for males; = 0.135 to 0.424 for females; all *p*-values < 0.001). Finally, the concurrent validity of the MISS-LG is shown in Table 4 with both HHRS-Homosexuality and AAQ measures. The correlations between the three factors of MISS-LG were strong in magnitude (*r* = 0.752 to 0.937 for males; = 0.640 to 0.784 for females; all *p*-values < 0.001). Moreover, the three MISS-LG factors were positively associated with HHRS-Homosexuality scores (*r* = 0.220 to 0.256 for males; = 0.190 to 0.384 for females; all *p*-values < 0.001) and AAQ scores (*r* = 0.323 to 0.427 for males; = 0.135 to 0.424 for females; all *p*-values < 0.001).

## 4. Discussion

This study used various statistical methods to examine the psychometric properties of a Chinese version of the MISS-LG. The results derived from CFA and Rasch analysis all suggest that the traditional Chinese version of the MISS-LG had satisfactory construct validity across groups. Specifically, CFA results indicated that the factorial structure of the MISS-LG included three factors in both male and female sexual-minority individuals in Taiwan. Therefore, the sexuality, identity, and social discomfort factors are different constructs in the MISS-LG, which echoes findings from Lingiardi et al. [26]. Rasch analysis additionally suggested that each factor was unidimensional: the items under the same factor of the MISS-LG were grouped together to demonstrate the same constructs. The concurrent validity of the MISS-LG was also supported by the significant correlations of the three MISS-LG factor scores with HHRS-Homosexuality and AAQ scores.

The CFA results indicated that most items had an acceptable factor loading. However, we found that item 11 for males (“If you are gay, it is better to have an “active” sexual role”) had a low factor loading (factor loading = 0.167). Therefore, item 11 might not well adhere to its embedded sexuality factor. The sexuality factor indicates the negative attitudes toward sexual behaviors and intimate relationships in lesbian women, gay men, or bisexual individuals. However, item 11 describes a value judgment on a specific sexual behaviors or positions rather than sexual behaviors or intimate relationships in gay men. This may be a possible reason to explain the low factor loadings for item 11 among males. It is also possible that cultural differences relating to individualism versus collectivism in Western and Eastern cultures may in part underlie apparent differences. Another item for males was found to have slightly low factor loadings; specifically, item 8 for males (“I do not believe in love between homosexual individuals”; factor loading = 0.270). Item 8 for males does not explicitly describe attitudes toward sexual behaviors or intimate relationships. For example, whether a person “believes in love” could be influenced by past experiences of dating. Therefore, when a person reads this sentence, the focus may not be on the intimate relationship of the individual per se. One more item, item 11 for females, also had a factor loading lower than 0.3 (factor loading = 0.299). However, this item (i.e., “Lesbians can only have flings/one-night stands”) conceptually fits well in the sexuality factor and its factor loadings is extremely close to 0.3. Nevertheless, future studies are needed to further explore the factor structure of MISS-LG for both the male and female versions.

The three factors of the MISS-LG were positively associated with HHRS-Homosexuality and AAQ scores. A high HHRS-Homosexuality score indicates a high level of perceived stigma regarding homosexuality from families. Family is the microsystem in which individuals have typically lived since childhood [47]. LGB individuals who perceive stigmatizing attitudes toward homosexuality may experience long-lasting impacts on their self-identity and internalize perceived stigma. A high AAQ score indicates a low level of psychological flexibility. Individuals with low psychological flexibility lack adaptable perspectives to observe and think without attachment to particular experiences, and therefore they may become trapped by past experiences and find it difficult to move forward [48]. LGB individuals who have low psychological flexibility may lack the effort to challenge social sexual stigma and internalize it as self-stigma. The significant correlations between MISS-LG and HHRS-Homosexuality and AAQ scores support the concurrent validity of the MISS-LG.

There are several limitations in this study. First, participants were a group of LGB individuals in early adulthood; thus, our results may not generalize to other age groups. Second, biases inherent to questionnaires (e.g., relating to social desirability) should be considered. Although we assured participants that all questionnaires were anonymous and individual data would be kept confidential, we cannot guarantee that every participant had no other concerns when they completed the questionnaires. Third, we did not conduct test–retest reliability and responsiveness for the MISS-LG. Therefore, it is unclear whether the traditional Chinese version of the MISS-LG has good reproducibility over time and whether it is sensitive to detecting changes in self-stigma. Fourth, the study included only lesbian, gay, and bisexual individuals. How sexual stigma may impact other sexual minority groups (e.g., transsexual, asexual) should be considered in future studies. Fifth, this study inquired participants’ gender identities by the binary of male and female but did not include the options of transgender, gender nonbinary, or genderqueer. Research has found that sexual and gender minority identities have intersectional impacts on health [49] and behaviors [50]; both sexual and gender minority identities should be considered in public health practice [51].

## 5. Conclusions

In conclusion, the MISS-LG has relatively satisfactory psychometric properties in a sample of LGB individuals in Taiwan. The three factors of the MISS-LG were confirmed in the CFA results; however, two items (Item 8 in male participants; Items 8 and 11 in both males and females) were found to have relatively low factor loadings in the sexuality factor. Each factor of the three MISS-LG factors were found to be unidimensional and most items fit satisfactorily in their embedded constructs, except for slight misfitting of Item 3 and concurrent validity of the MISS-LG was supported by the positive correlations with HHRS-Homosexuality family subscale and AAQ scores. However, more investigation is needed to understand reasons for the slight misfitting of several MISS-LG items.

## Figures and Tables

**Table 1:** Participants’ characteristics (*N* = 1000).

Variables	Male (*N* = 500)	Female (*N* = 500)
Age in years; M (SD)	24.80 (2.91)	24.45 (3.06)
Educational level; *N* (%)		
High school or below	64 (12.8)	45 (9.0)
College or above	436 (87.2)	455 (91.0)
Sexual orientation; *N* (%)		
Homosexual	365 (73.0)	205 (41.0)
Bisexual	135 (27.0)	295 (59.0)
Paternal education; *N* (%)		
High school or below	319 (63.8)	272 (54.4)
College or above	181 (36.2)	228 (45.6)
Maternal education; *N* (%)		
High school or below	355 (71.0)	305 (61.0)
College or above	145 (29.0)	195 (39.0)
Sexual orientation known by family		
No or few	383 (76.6)	398 (79.6)
Many or a great quantity	117 (23.4)	102 (20.4)
Sexual orientation known by friends		
No or few	195 (39.0)	159 (31.8)
Many or a great quantity	305 (61.0)	341 (68.2)
Sexual orientation known by online friends		
No or few	187 (37.4)	289 (57.8)
Many or a great quantity	313 (62.6)	211 (42.2)

**Table 2:** Confirmatory factor analysis results and item properties for the Measure of Internalized Sexual Stigma for Lesbians and Gay Men (MISS-LG).

Items		Male				Female		
	Factor Loading	Mean (SD)	Skewness	Kurtosis	Factor Loading	Mean (SD)	Skewness	Kurtosis
Sexuality								
I2	0.751	2.89 (1.16)	0.07	−0.94	0.542	1.74 (0.90)	1.24	1.16
I5	0.466	1.85 (1.03)	1.11	0.51	0.479	1.42 (0.83)	2.12	3.79
I8	0.270	1.45 (0.79)	1.97	3.87	0.499	1.12 (0.36)	3.13	9.77
I11	0.167	2.99 (1.01)	−0.01	−0.23	0.299	1.24 (0.57)	2.87	9.46
I14	0.390	1.76 (1.00)	1.25	0.85	0.380	1.33 (0.64)	2.15	4.61
Identity								
I3	0.604	2.06 (1.16)	0.86	−0.29	0.725	1.65 (0.84)	1.27	1.25
I6	0.741	1.81 (1.08)	1.17	0.48	0.568	1.47 (0.76)	1.62	2.34
I9	0.758	2.41 (1.21)	0.34	−0.87	0.635	2.24 (1.21)	0.52	-0.96
I12	0.727	1.80 (0.94)	0.89	−0.22	0.713	2.02 (1.02)	0.63	-0.43
I15	0.748	2.65 (1.37)	0.20	−1.25	0.828	1.65 (0.86)	1.31	1.17
Social discomfort								
I1	0.710	2.50 (1.16)	0.37	−0.77	0.609	2.82 (1.02)	-0.11	−0.64
I4	0.723	2.52 (1.17)	0.31	−0.85	0.713	1.80 (0.95)	1.08	0.40
I7	0.611	2.63 (1.18)	0.22	−1.00	0.753	2.50 (1.19)	0.40	−0.91
I10	0.778	2.54 (1.16)	0.31	−0.87	0.798	2.10 (1.04)	0.72	−0.26
I13	0.757	2.90 (1.29)	-0.02	−1.16	0.566	2.03 (1.01)	0.71	−0.27
I16	0.670	2.76 (1.25)	0.06	−1.11	0.697	1.96 (1.09)	0.86	−0.35
I17	0.428	2.39 (1.13)	0.35	−0.78	0.721	1.67 (0.87)	1.34	1.39

**Table 3:** Rasch analysis results for the Measure of Internalized Sexual Stigma for Lesbians and Gay Men (MISS-LG).

Items		Male			Female	
	Difficulty	Infit	Outfit	Difficulty	Infit	Outfit
Sexuality						
I2	−0.86	0.91	0.91	−0.66	1.11	1.03
I5	0.40	1.00	1.00	−0.04	0.85	0.75
I8	0.97	0.92	0.80	0.60	0.80	0.72
I11	−1.03	1.36	1.36	0.37	1.05	1.07
I14	0.53	0.76	0.73	−0.26	1.20	1.18
Identity						
I3	0.00	1.44	1.53	0.37	0.99	1.06
I6	0.43	0.81	0.80	0.85	1.16	1.24
I9	−0.54	0.85	0.84	−1.12	1.01	1.01
I12	1.12	0.88	0.87	−0.54	0.91	0.91
I15	−1.01	1.01	0.98	0.44	0.93	0.93
Social discomfort						
I1	0.11	0.92	0.96	−1.29	1.07	1.06
I4	0.12	0.84	0.85	0.72	0.98	0.98
I7	−0.02	1.10	1.14	−0.82	0.94	0.93
I10	0.08	0.88	0.88	0.01	0.99	0.96
I13	−0.45	0.82	0.79	0.22	1.36	1.47
I16	−0.21	0.96	0.96	0.23	0.88	0.86
I17	0.36	1.45	**1.51**	0.93	0.82	0.68

Infit = infit mean square; outfit = outfit mean square; values in bold indicate misfit (i.e., >1.5).

**Table 4:** Concurrent validity results for the Measure of Internalized Sexual Stigma for Lesbians and Gay Men (MISS-LG).

Variables	MISS-LG Sexuality	MISS-LG Identity	MISS-LG Social Discomfort	HHRS-Homosexuality	AAQ
MISS-LG Sexuality	(0.67/0.64)	0.752 ***	0.937 ***	0.220 ***	0.427 ***
MISS-LG Identity	0.784 ***	(0.87/0.87)	0.784 ***	0.231 ***	0.339 ***
MISS-LG Social discomfort	0.700 ***	0.640 ***	(0.90/0.91)	0.256 ***	0.323 ***
HHRS-Homosexuality family	0.190 ***	0.270 ***	0.384 ***	(0.95/0.94)	-
AAQ	0.287 ***	0.282 ***	0.424 ***	-	(0.94/0.95)

Abbreviations: Measure of Internalized Sexual Stigma for Lesbians and Gay Men (MISS-LG); HIV and Homosexuality Related Stigma (HHRS); Acceptance and Action Questionnaire-II (AAQ). Upper triangular matrix reports correlation coefficients from male participants; lower triangular matrix reports correlation coefficients from female participants; diagonal values are McDonald’s omega, where the first values derived from male participants and the second values from female participants. *** *p* < 0.001.

## Data Availability

The data will be available upon reasonable request to the corresponding authors.
